# Treatment planning for very high energy electrons: Studies that indicate the potential of the modality

**DOI:** 10.1016/j.phro.2024.100670

**Published:** 2024-11-06

**Authors:** James L. Bedford, Uwe Oelfke

**Affiliations:** Joint Department of Physics, The Institute of Cancer Research and The Royal Marsden NHS Foundation Trust, London SM2 5PT, United Kingdom

**Keywords:** Very high energy electrons, Treatment planning, Radiotherapy, Penumbra, Beam energy, FLASH

## Abstract

**Background and purpose:**

Radiotherapy using Very High Energy Electrons (VHEE) has the potential to reduce dose to organs at risk compared to photons. This article therefore reviews treatment planning for VHEE, to clarify the potential benefit of the modality.

**Materials and methods:**

Articles on VHEE were identified and those which focused on treatment planning were manually selected, particularly those which contained results on patient datasets. Benefits in absorbed dose to organs at risk were converted to percentages of prescription dose so as to provide uniform, clinically relevant reporting.

**Results:**

Increased beam energy was found to reduce electron scatter and give rise to a narrower penumbra but lead to a rather constant depth dose curve, which was not as useful for sparing normal tissues as that of protons. The sharp penumbra of VHEE was of benefit in treatment planning for producing treatment plans with conformal dose shaping, with improved dose to critical structures being demonstrated for several treatment sites. Mean dose to critical structures, relative to the prescribed dose, was in the order of 0–10% lower than photons and 0–10% higher than protons. The delivery technology and dose distributions were also promising for radiotherapy with ultra-high dose rate (FLASH).

**Conclusion:**

At present, the potential clinical benefit of VHEE relative to photons or protons is small. Further studies are needed to more precisely quantify the relative performance of broad beams versus pencil beam scanning and to investigate treatment sites that might benefit maximally from the use of VHEE beams.

## Highlights

• Very high energy electrons (VHEE) may increase conformality of radiotherapy.
• This article reviews treatment planning studies to quantify this benefit.
• VHEE provides a small reduction in dose to organs at risk compared to photons.
• VHEE gives a higher dose to organs at risk compared to protons.
• There may be specific applications such as exploiting ultra high dose rate.

## 1 Introduction

The field of Very High Energy Electrons (VHEE) covers electrons in the energy range from 50 MeV to 300 MeV [1], with most studies being in the range 100 MeV to 250 MeV. Electrons with energies higher than this have been referred to as Ultra High Energy Electrons (UHEE) [1] with at least one study investigating an energy of 2 GeV [2]. At the 250-MeV upper limit of VHEE, the particles pass through the patient, so it is unlikely that a further increase beyond this energy has a practical benefit, although, as with much of this field, this is as yet unproven.

Ideally, therapy treatment beams should be conformal to the planning target volume (PTV) both proximally and distally, as well as laterally. Photons are the most common particles in use for therapy today, but these give a high dose to the tissue proximal to the PTV, and a lower dose to the tissue distal to the PTV. Laterally, there is a penumbra whose 80 % – 20 % width is of the order of 5 mm [3]. Protons are increasingly being used for high-quality therapy. These have a relatively high entrance dose, but negligible exit dose. Laterally, the penumbra is slightly narrower than with photons but may be broader at the Bragg peak itself [4]. Electron beams can potentially provide similar quality beams to protons [5]. The goal of electron therapy is to imitate the low entrance and exit dose of protons, but in practice, the entrance dose is approximately that of protons, while the exit dose is much higher than that of protons. Moreover, the penumbra of a VHEE beam can be very narrow, depending on energy and field size [6].

As well as the beneficial characteristics of the VHEE depth dose curves themselves, further clinical benefit may be obtained by increasing the dose rate of the beams to an ultra-high rate. The resulting short delivery time is resilient to intra-fraction motion effects, with the treatment being completed in a very small portion of the breathing cycle. Of greater interest, if the dose rate is increased above approximately 40 Gys^−1^, with a dose per fraction of at least 10 Gy and an overall fraction delivery time of less than 0.1 s [7], [8], [9], the so-called FLASH effect should be observed in normal tissues, in which the normal tissue reaction to radiation is lower than with current clinical dose rates of 0.1 Gys^−1^ [10], [11], [12], [13], [14]. There is an additional practical motivation for using VHEE at ultra-high dose rate (UHDR) in that many of the VHEE delivery systems available are designed to operate at such high dose rates, with fast beam scanning systems, giving them an operational advantage over clinical systems used for delivery of photons or low-energy electrons.

The field in general, including the laboratory generation of VHEE beams, has been reviewed by Ronga et al [15]. However, it was clear that it was the outcome of the treatment planning work which held the key to determining which methods were most likely to be of clinical benefit, and therefore which areas should be addressed with the greatest priority. The present review therefore concentrated on the work that has been accomplished in the field of treatment planning for VHEE and aimed to make an overall objective assessment of the resulting sparing of critical structures.

## 2 Method and materials

The study was conducted by following up references in the key treatment planning papers of which the authors were aware. Other articles were also selected to give sufficient background and motivation to the review. To verify the results of this selection, the PubMed and Web of Science databases were searched for articles containing “VHEE” or “Very High Energy Electrons” in the title and the results examined for treatment planning studies. This review was then structured as follows: the results of Monte Carlo simulations of simple beams were outlined as giving the basis for any observed benefits in treatment planning. Practical implementation of VHEE was also described briefly as this formed the background to the rest of the review and contained some important lessons to be learned. The body of the review then gave a chronological outline of the key treatment planning studies which indicated the potential of VHEE.

The findings of these studies were tabulated for convenient review. The individual tables and figures of the papers were used for this purpose. In particular, many of the studies reported dose sparing with VHEE relative to the dose delivered by a comparative technique such as photons or protons. This method gave results which were highly dependent on the dose given by the comparative technique, so in this review, all results were converted to percentages of the prescribed dose. Similarly, changes in volume were converted to percentages of the total organ volume. This led to differences from the values reported in the abstracts of the original articles, but was considered to be a more consistent and clinically representative approach. In the interests of brevity, statistics that were similar for both VHEE and comparative plans were not reported.

## 3 Results

### 3.1 Historical Monte Carlo studies of simple beams

One of the earliest studies of VHEE in the context of radiation therapy was that of Des Rosiers et al [16]. They used the PENELOPE Monte Carlo code [17] to simulate the dose distribution from broad and stereotactic beams. They found that beams with energy 150 MeV to 250 MeV had a role to play in the provision of a high dose to the PTV and minimal dose to normal tissues, particularly if the energy of the beam could be tailored to the depth of the PTV, so that the maximum dose in the depth dose curve could be arranged to coincide with the depth of the PTV. The 90 % − 20 % penumbra width at 100 mm depth in phantom varied from 7.9 mm to 6.1 mm as energy varied from 150 MeV to 250 MeV. These authors also concluded that some form of pencil beam scanning might be used to provide effective intensity modulated radiation therapy (IMRT).

Papiez et al. [18] also showed that for energies of greater than 200 MeV, the penumbra width of a VHEE beam was comparable with that of a 10 MV photon beam. They also showed the impact of delivering a VHEE beam through an air-density region. With 15 MV photons, the beam scattered significantly at the region of low tissue density, resulting in a broadening of the penumbra by around 20 mm and a loss of dose in the beam of up to 50 %. Meanwhile, the 200 MeV electrons passed across this region with negligible scatter, so that no broadening of the penumbra was observed.

### 3.2 Recent Monte Carlo studies of simple beams

Bazalova-Carter et al. [19] demonstrated agreement between Monte Carlo simulations and measurements using radiochromic film for beams of 50 MeV to 70 MeV energy. The beams had full width at half maximum (FWHM) of 4 mm to 7 mm at 6.4 mm depth in the phantom and the agreement between simulations and measurements was broadly better than 5 %.

**Fig. 1:**
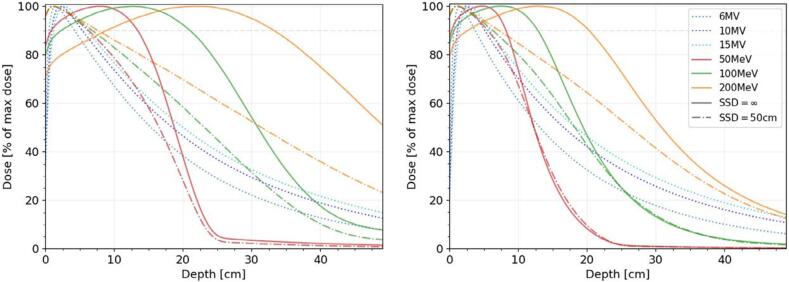
Depth dose curves for photons and high energy electrons at 500 mm source to surface distance (SSD) and infinite SSD. Left: 100 mm x 100 mm field, right 30 mm x 30 mm field. Reproduced from Böhlen et al. [6] with permission. © 2021 American Association of Physicists in Medicine.

The work of Böhlen et al. [6] set out comprehensively the characteristics of VHEE beams of various energies. These authors used the Monte Carlo electron algorithm in the RayStation treatment planning system (RaySearch Laboratories, Stockholm, Sweden) to calculate dose distributions for simple beam combinations and compare the results with measurements, GEANT4 simulations [20] and PENELOPE simulations [17] (Fig. 1). For beams with 50 to 250 MeV and source to surface distance (SSD) of 1000 mm or more, the therapeutic range, defined as the depth of penetration to 90 % of maximum dose, increased almost linearly with energy. Due to the curved shape of the depth dose curve, adding a parallel opposed beam was of little benefit. Penumbra was found to be generally broader than with photon beams for field widths of 30 to 100 mm, although at energies of greater than 200 MeV, the difference was not very marked at depth and in favour of electrons at shallower depths of 50 mm to 150 mm.

### 3.3 Beam focusing

**Fig. 2:**
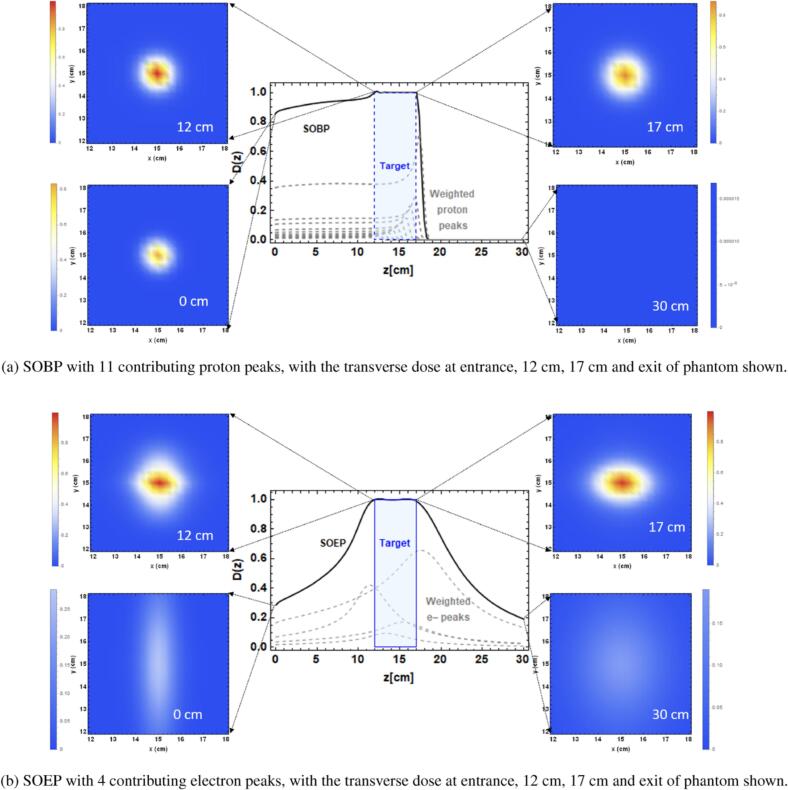
Monte Carlo simulations of dose distributions in a water phantom using TOPAS. (a) Spread-out proton Bragg peak (SOBP) and (b) spread-out electron peak (SOEP) produced from focused 250 MeV VHEE beams in which quadrupole strength is varied to shift the peak position. In both cases, the transverse dose cross sections are shown at the entrance, beginning of the target, end of the target and exit. Reproduced from Whitmore et al. [21] under CC BY 4.0 license http://creativecommons.org/licenses/by/4.0/.

The aim here was to direct the electron fluence into the patient in a convergent manner, so as to avoid the increase in penumbra width seen in the previous studies, and to improve the characteristics of the depth dose curve. Whitmore et al. [21] simulated the behaviour of narrow focused VHEE beams using the TOPAS Monte Carlo code [22]. By focusing the Gaussian beam using six quadrupole magnets, the depth dose curve could be adjusted so that it peaked at a particular depth in a phantom. The peak was considerably more spread out over depth than the Bragg peak of a proton beam (Fig. 2), but nevertheless, a “spread out electron peak” (SOEP) could be created. This peak gave a much lower integral dose to the normal tissue proximal and distal to the PTV.

Kokurewicz et al. [23] demonstrated an alternative form of beam focusing. They used a broad beam with a FWHM in the order of 200 mm and focused the beam with an ideal magnetic lens, with a focal length chosen so that the beam focused at the depth of the PTV. The approach irradiated normal tissue to lower doses, with lower entrance and exit doses than collimated electron beams. The practical possibility of this method was demonstrated using Monte Carlo simulations with the FLUKA code [24], [25] and using EBT3 film (Ashland, Wilmington, DE) [26], [27].

### 3.4 Practical realisation

A clinical VHEE treatment unit has already been developed and implemented [28]. The MM50 unit was a racetrack microtron with beam energy up to 50 MeV in steps of 5 MeV. Although this was at the lower extreme of the energy range of interest for VHEE, its application was relevant. The machine was equipped with a multileaf collimator consisting of 32 pairs of 12.5 mm leaves. Although patients were treated with this device, it did not find widespread use and is no longer available.

### 3.5 Historical treatment planning simulations

The simulation study of Asell et al. [29] used electrons in the range 5 to 30 MeV, and therefore did not really rank in the VHEE category, but showed how fluence and energy of the electron beam could be modulated to overcome the effects of inhomogeneities in the patient. A further study using energies of up to 100 MeV [30] considered cranial and cervical targets and used several electron beams either alone or in combination with photon beams to create conformal treatment plans with high probability of uncomplicated tumour control (P+).

The racetrack microtron described in the previous section was the motivation for the work of several authors. Karlsson and Zackrisson described the use of electrons with energy up to 50 MeV to treat deep targets, with orthogonal wedged photon beams being used to compensate for the gradual distal fall-off of the electron beam [31]. Mixing the electron beam with a photon component of up to 50 MV was also shown to reduce the high skin dose of the electron technique. These authors also described the application of pencil beam scanning on the MM50 racetrack microtron to producing simple beam patterns such as wedges [32]. Lief et al. used the scanned beam on the MM50 device to provide optimal dose profiles at various depths in the patient [33]. This was particularly applied to sharpening the beam penumbra, for lateral sparing of critical structures.

Hyodynmaa et al. [34] optimised the fluence intensities of a series of incident beams of different energies, in a technique that has become widely used in proton pencil beam scanning. Ebert and Hoban [35] also evaluated the possibility of optimising beam angle, pencil beam position and beam energy simultaneously, in a potentially very useful manner. Electron beams with energies of up to 45 MeV were combined with photon beams by Korevaar et al. [36] for a series of clinical cases with a view to improving target coverage and reducing dose to organs at risk.

These studies were below the energy threshold of VHEE, but they were important in highlighting what might be achievable with electron beams. In contrast, the work of Yeboah et al. [37] related to VHEE directly, using a two-dimensional computational phantom to mimic a prostate volume and finding that a beam energy of greater than 100 MeV was desirable, with between nine and 21 equally spaced beams providing acceptable dose distributions. Use of beam orientation optimisation influenced these plans even when using as many as 15 beams. Fewer than nine beams could be used if approximately three beam energies were used at each of the beam angles. Use of rotation therapy was found to increase the dose to normal tissues compared to 21 fixed beam orientations.

These authors also compared VHEE at a beam energy of 250 MeV with photons of energy 15 MV and protons of energy 200 MeV [4]. Between five and 11 beams were used for VHEE and photons, while one to nine beams were used for protons. Two-dimensional simulations of prostate target volumes were used to show that in comparison with photons, VHEE reduced bladder and rectum dose by around 10 %. A corresponding comparison of protons with photons showed that bladder and rectum dose could be reduced by around 30 %. The benefit of VHEE was largely due to its reduced penumbra width, while that of protons was due additionally to the low distal dose.

### 3.6 Recent treatment planning simulations

The first reported study of VHEE using three-dimensional treatment planning was that of DesRosiers [38]. This study used Monte Carlo simulation of 150–250 MeV beams in conjunction with CT data for radiotherapy of prostate cancer. The authors used the beam parameters of a laser-plasma wakefield accelerator [39], [40], [41] to produce realistic treatment plans, concluding that the dose distributions with VHEE were similar to those of photons or protons, but with advantages in terms of beam delivery compared to these other modalities. As well as using CT data for treatment planning, the study was also pioneering in its application of real beam parameters from a novel accelerator.

The work of Fuchs et al. [42] considered the application of laser-accelerated VHEE beams to treatment planning with beam energies of 150 MeV, 185 MeV and 250 MeV and with an FWHM of the generating energy peak of approximately 15 MeV. Practically obtained beam parameters were used as a phase space for Monte Carlo simulations using GEANT4 [20]. The FWHM for the lateral dose distribution at 100 mm depth was approximately 10 mm. The Monte Carlo simulated dose distributions were used to calculate the dose-influence matrices for inverse planning, which were then applied to a prostate case. The results were compared with a photon intensity-modulated radiation therapy (IMRT) inverse plan produced using the same optimisation scheme. The authors reported improved target coverage with the 250 MeV VHEE plans delivered at 1000 mm SSD compared to a 6 MV photon plan. Mean doses to rectum and bladder were reduced with VHEE due to sharper penumbra. A similar study was also reported by Moskvin et al. [43] at around the same time, using a Monte Carlo treatment planning system based on the PENELOPE code [17].

**Fig. 3:**
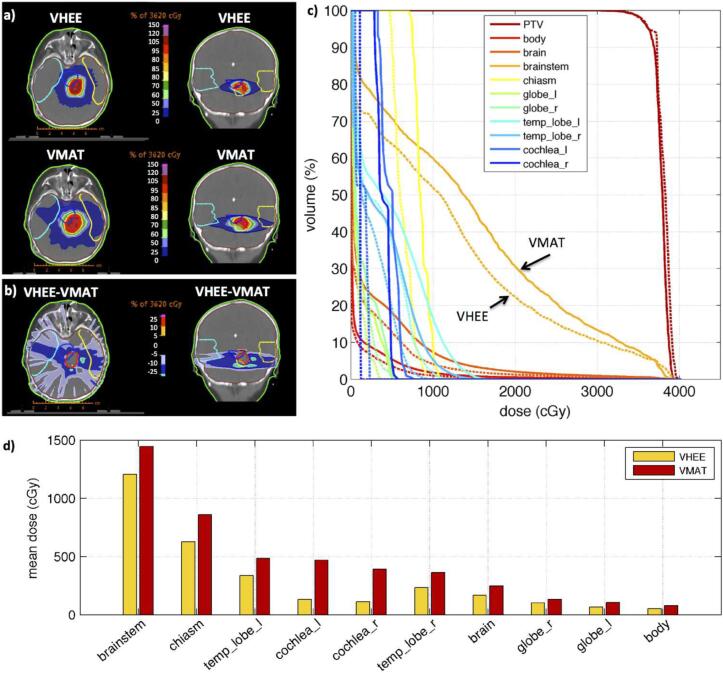
An example paediatric brain case showing (a) dose distributions, (b) dose differences, (c) dose-volume histograms and (d) comparative statistics. Reproduced from Bazalova-Carter et al. [44] with permission. © 2015 American Association of Physicists in Medicine.

Some very informative studies were also reported by Bazalova-Carter et al. [44] for a variety of clinical tumour sites. They used the EGSnrc Monte Carlo code [45] in conjunction with RayStation to produce treatment plans for a pencil-beam scanning technique. The beam energy for this technique was between 60 MeV and 120 MeV and the FWHM was between 0.1 and 5.0 mm. The source to axis distance (SAD) was 400 mm for these cases. For a paediatric brain case, VHEE with 36 beams of 100 MeV compared favourably with the volumetric modulated arc therapy (VMAT) treatment plan consisting of 6 MV photons used for clinical treatment of the patient (Fig. 3). This was principally due to the greater conformality of the VHEE treatment plan around the PTV. The result was that the integral dose to brain, brainstem and other critical structures was reduced with the VHEE plan.

Similar sparing of critical structures was also observed in a lung case [44], where irradiated volume of the ipsilateral lung was reduced, compared to a single-arc VMAT plan with a 6 MV flattening filter-free (FFF) beam. For prostate, the difference between VHEE and VMAT was less clear, with bladder showing the greatest sparing using VHEE compared to a dual arc VMAT plan with 15 MV beams, but this was not particularly marked. The conclusion of this treatment planning study was that 36 VHEE beams of 100 MeV were able to provide competitive treatment plans with respect to photon treatment plans as used in current clinical practice.

**Fig. 4:**
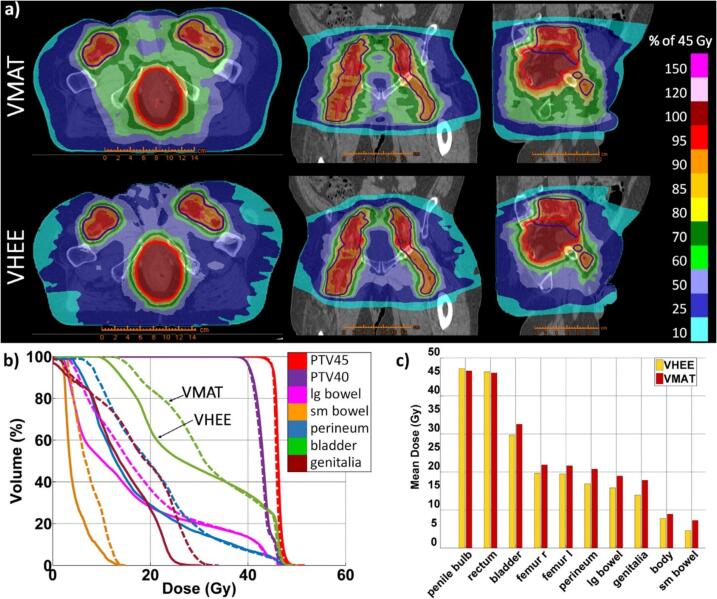
A comparison of three-arc 15 MV volumetric modulated arc therapy (VMAT) and 32-beam 100 MeV very high energy electrons (VHEE) for an anal cancer patient. (a) dose distributions, (b) dose-volume histograms and (c) comparative statistics. Reproduced from Palma et al. [46] with permission. © 2016 Elsevier Ireland Ltd. All rights reserved.

A similar study, based on more realistic accelerator output, was described by Palma et al. [46]. They retrospectively planned five clinical cases, consisting of acoustic neuroma, liver, lung, oesophagus and anus, using beam energy of 100 MeV and 120 MeV. The scanning pencil beams had a FWHM of 1–5 mm and dose was calculated using BEAMnrc and EGSnrc. The treatment plans consisted of 16 or 32 equally spaced coplanar beams and these were compared with Cyberknife (Accuray Inc., Sunnyvale, CA), which had a beam energy of 6 MV, and VMAT plans with energy between 6 and 15 MV. Over all of the cases, the PTV coverage was similar between the VHEE and VMAT plans, whereas the VHEE plans provided a lower mean dose to the critical structures than VMAT (Fig. 4). Integral dose to the whole body was also lower than with VMAT. Use of scanned electron pencil beams was therefore shown to be an attractive alternative to current photon treatment techniques.

**Fig. 5:**
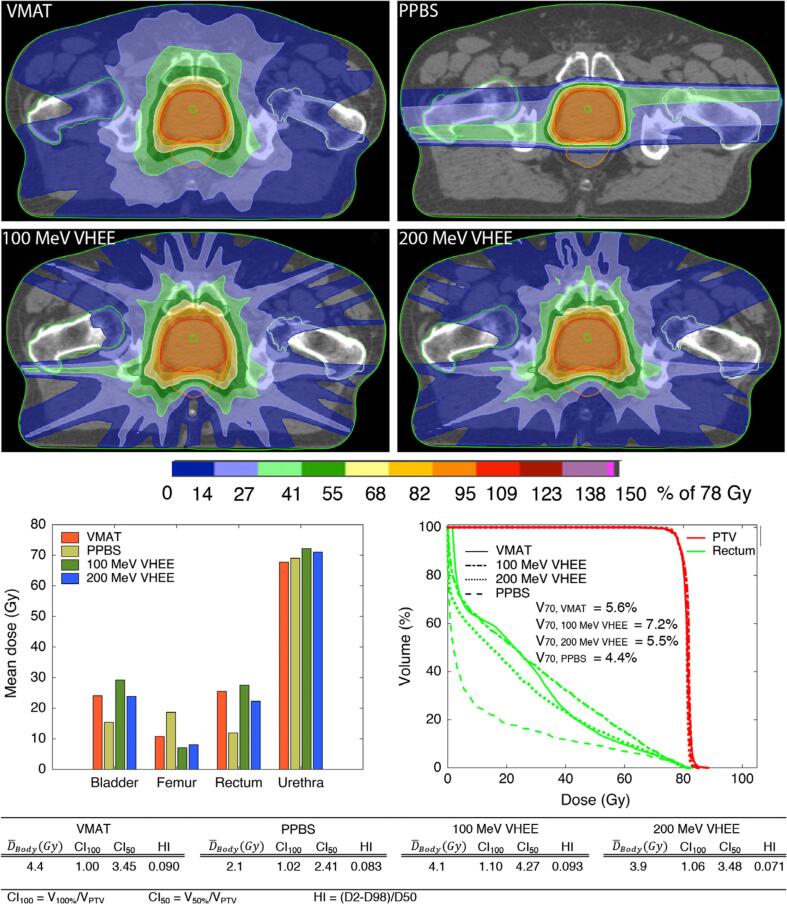
A comparison of volumetric modulated arc therapy (VMAT), very high energy electrons (VHEE) and proton pencil beam scanning (PPBS) for prostate, planned by an automated algorithm. CI: conformity index, HI: homogeneity index. Reproduced from Schüler et al. [47] with permission. © 2017 American Association of Physicists in Medicine.

The same group of authors [47] also used a similar planning arrangement within RayStation to optimise four clinical plans (prostate, lung, paediatric brain and head and neck) for scanning electron beam therapy and to compare them with VMAT and proton pencil beam scanning (Fig. 5). The focus of this work was to use a planning framework which reduced operator bias during the treatment planning comparison. The results broadly showed that, for equivalent PTV coverage, the proton plans showed the lowest dose to critical structures, followed by the scanning electron beam plans and then photon VMAT plans.

Three prostate cases planned using VHEE beams of energy between 70 MeV and 130 MeV were also examined by Sarti et al. [48] in comparison with IMRT plans of between five and seven beams. The FLUKA Monte Carlo code was used for this comparison [24], [25]. The approach in this study was to adjust the scanned pencil beams in energy so that the dose maximum coincided with the depth of the PTV, thereby giving optimal normal tissue sparing. The method was shown to produce results that were favourable with respect to IMRT.

### 3.7 Ultra high dose rate (FLASH) utilisation

The works of Böhlen et al. explored the characteristics of VHEE treatment plans for potential application to UHDR delivery [49], [50]. The first of these [49] considered seven glioblastoma and seven lung cases in comparison with clinical VMAT or helical tomotherapy treatment plans. Between three and 16 equally spaced VHEE beams were used, with energy of either 100 MeV or 200 MeV. A couch rotation of up to 30° was allowed for the glioblastoma cases. Unlike previous studies which used intensity-modulated beams, this study used simple unmodulated beams so that the overall treatment time could be kept to 100 ms, for application to UHDR treatment, with dose calculation provided by the RayStation electron Monte Carlo algorithm, based on VMC++, extended to higher energies. The FLASH sparing effect was not in itself modelled by this study. The results showed similar dose statistics for VHEE as for clinical photon treatments.

The second work [50] modelled scanned VHEE pencil beams using a new dose calculation algorithm within RayStation. Here the focus was on comparison with transmission proton beams, for potential application to UHDR delivery, and the same optimisation framework was therefore used for both VHEE and proton optimisation. Glioblastoma, oesophagus and prostate cases were considered. In general, the transmission proton treatment plans gave the best sparing of critical structures and normal tissues, followed by 200 MeV electrons, with 100 MeV electrons being less effective in sparing. These results were consequent upon a trade-off between depth dose curves and penumbra width. At higher energies, the depth dose of the VHEE beam was moderately flat, similar to the proximal part of the proton beam depth dose, but the penumbra width was lower than at lower energies, although still larger than with protons. Another study of note was that of Breitkreutz et al. [51], who also used simple parallel-opposed 40 MeV electron beams for a paediatric brain case to achieve FLASH dose rates.

The study of Sarti et al. [48] referred to in section 3.6 investigated the potential impact of UHDR delivery. By applying dose modifying factors to represent reduced biological response in the normal tissues, these authors were able to show very competitive dose distributions in relation to clinical photon treatments. The dose modifying factors were almost certainly more complex in reality than the simple model used here, because the factors depended on delivered dose and dose rate, both of which were spatially variable around the treatment site. The study also assumed 2 Gy fractions, in which the FLASH effect was not expected to be exhibited. Nevertheless, the study showed promising results for VHEE in conjunction with ultra high dose rate.

A more comprehensive model of the effect of UHDR delivery was used by Zhang et al. [52]. These authors used a scanning pencil beam approach to VHEE and a simple timing model to calculate the average dose rate and dose-weighted average dose rate. The FLASH effect could therefore be calculated more accurately, within the limits of the accuracy of the model itself. However, the applicability of dose-averaged dose rate was not well validated and did not appear from other studies to be well correlated with FLASH effect [8], [53]. The results of this study showed dose-averaged dose rates of approximately 100 Gys^−1^ but total irradiation times for 10 Gy fractions of around 1–5 s, which may have been too long for observation of the FLASH effect. However, what was particularly exciting about this study was the application of the method to relatively superficial brain and lung tumours, where electron treatments were mostly likely to succeed. By using 11–16 beams arranged around the proximal side of the PTV, the authors of the study were able to create dose distributions that resembled those of VMAT arcs.

The Pluridirectional High-energy Agile Scanning Electronic Radiotherapy (PHASER) system [54] used a scanned narrow electron pencil beam impinging on a fixed target to deliver photons at ultra-high dose rate. There was also scope to remove the fixed target from this device and to increase the electron beam energy, so as to deliver VHEE therapy at sufficiently high dose rate for practical FLASH therapy. The reader is referred to Ronga et al. [15] for a review of the use of VHEE beams particularly in the context of FLASH radiotherapy.

The study of Muscato et al. [5] compared both proton and photon therapy with VHEE for meningioma and chordoma cases and concluded that VHEE was able to compete with both of the alternative state-of-the-art techniques. The potential impact of FLASH delivery was then included, showing that dose could be further optimised when using ultra-high dose rate. In this study, as with all studies of UHDR, a high dose per fraction was needed to exhibit the FLASH effect, and the practical use of such a high dose per fraction may not be possible. The area of research at the interface between VHEE and UHDR is reviewed by Rahman et al. [55], while Table 1 summarises the main treatment planning studies of VHEE to date, together with their key findings.

**Table 1:** Summary of VHEE treatment planning studies.

Study/authors	Tumour site(s)	VHEE technique*	Beam energy (MeV)	Comparative technique*	Statistic considered	Percent benefit of VHEE†□
Muscato et al. [5]	Meningioma, chordoma	3–7 PBS beams	60–120	IMRT, protons	PTV V95% and various mean and D1 OAR doses	Comparable to IMRT, 0–10 % worse than protons
Asell et al. [30]	Cervix, brain simulations	Single IMB + extra IMB and photons	25–100	Single IMB e- beam	P+	20 %
Karlsson and Zackrisson [31]	Mediastinum, neck, pelvis, oesophagus	Conformal	7–50	Photons	Lung V50%	10 % of total lung volume
Karlsson et al. [32]	Prostate	PBS wedges with extra 20 MV photons	50	50 MV photons	Rectum, bladder, femoral heads mean dose	Small for rectum and bladder, larger for femoral heads
Korevaar et al. [36]	Bladder, pancreas, chordoma, breast	IMB with photon IMRT	15–45	IMRT alone	Mean dose, TCP, NTCP	Marginal
Yeboah et al. [37]	Prostate simulation	IMB	50–250	50 MeV electrons	Rectum mean dose	15 % of prescribed dose
Fuchs et al. [42]	Prostate	PBS	150–250	6 MV IMRT	Rectum, bladder mean dose	5 % of prescribed dose
Bazalova-Carter [44]	Pediatric brain	PBS	60–120	6 MV VMAT	Brainstem, chiasm, cochlea, brain mean dose	5 % of prescribed dose
	Lung	PBS	100	6 MV FFF VMAT	Heart, oesophagus, lung, chest wall mean dose	2 % of prescribed dose
	Prostate	PBS	100	15 MV VMAT	Bladder, rectum, femurs mean dose	5 % of prescribed dose for bladder, less for the others
Palma et al. [46]	Anus	32 PBS beams	100	15 MV VMAT	Bowel, bladder, perineum, genitalia mean dose	10 % of prescribed dose
	Oesophagus	32 PBS beams	120	6 MV VMAT	Heart, liver, bowel, spinal cord mean dose	10 % of prescribed dose
	Acoustic neuroma	32 PBS beams	120	Cyberknife 6 MV photons (84 beams)	Cochlea mean dose	5 % of prescribed dose
	Lung	16 PBS beams	100	10 MV VMAT	Integral dose	Small
	Liver	32 PBS beams	120	10 MV VMAT	Integral dose	Marginal
Schüler et al. [47]	Prostate	16 PBS beams	100–200	6 MV VMAT and up to 225 MeV PBS protons	Bladder, rectum mean dose	Similar to VMAT and 10 % higher than protons
	Lung	16 PBS beams	100–200	6 MV VMAT and up to 225 MeV PBS protons	Oesophagus, lungs, bronchial tree mean dose	5 % better than VMAT, 5 % worse than protons
	Pediatric brain	16 PBS beams	100–200	6 MV VMAT and up to 225 MeV PBS protons	Parotid, chiasm mean dose	5 % better than VMAT, similar to protons
	Head and neck	16 PBS beams	100–200	6 MV VMAT and up to 225 MeV PBS protons	Spinal cord, parotid, oral cavity, brainstem mean dose	10 % better than VMAT, 5 % better than protons
Sarti et al. [48]	Prostate	5–7 PBS beams	70–130	6 MV IMRT with same directions as VHEE	Bladder and rectum V50Gy	3 % bladder, 10 % rectum
Böhlen et al. [49]	Brain	3–16 broad beams	100–200	6 and 10 MV VMAT, 6 MV tomotherapy	Brain, brainstem mean dose	Brain 0 %, brainstem 0–5 %
	Lung	3–16 broad beams	100–200	6 and 10 MV VMAT, 6 MV tomotherapy	Lung, heart, oesophagus mean dose	0 %
Böhlen et al. [50]	Brain, oesophagus, prostate	7 PBS beams	50–250	250 MeV PBS shoot-through protons	Various OAR irradiated volumes	0 %
Zhang et al. [52]	Brain, lung	11–16 PBS beams	80–140	No comparison	Ring structures	Clinically acceptable

*PBS: pencil beam scanning; IMB: intensity-modulated beams.

†Benefits are referred to prescription dose where possible. Many studies report benefit relative to original dose, which is already low, so percentage benefit appears high.

**□**In the interests of brevity, results are summarised. The reader is referred to the original studies (references in the left-hand column) for full details.

## 4 Discussion

The results show clearly that the reduction in mean dose to critical structures with VHEE in relation to photon IMRT or VMAT is in the order of 0–10 % of the prescribed dose. VHEE is dosimetrically inferior to proton therapy with pencil beam scanning, giving a higher mean dose to critical structures by around 0–10 % of the prescribed dose. Thus, the current position of VHEE is to provide an improvement in photon therapy, and to approach proton therapy without equalling it.

In a debate in 2004, Papiez argued that the best means of delivering image-guided 4D IMRT was to use scanned pencil beams and that VHEE was the best modality for this [56]. Bortfeld countered that image-guided therapy could be delivered with photons, that VHEE provided little benefit to 15 MV photons and that it was expensive to develop and implement. Twenty years later, it is interesting to reflect on this discussion. Scanned pencil beams are being used widely, but in the form of proton therapy and image-guided IMRT is also being used widely with photons. Both arguments have therefore been shown to be true to some extent. The real question, which is still open, is whether the benefit in the dose distribution of VHEE merits the cost of development of the new modality.

One potential area of application for VHEE is as a compact replacement for proton therapy. Due to the lower mass of the electrons compared to protons, the requirements for acceleration and steering are much more limited for VHEE. Consequently, implementation in a compact environment at lower cost may be possible. In this connection, use of lower energy beams, up to 100 MeV, may be beneficial and this is an area that few studies have explored. The interest in FLASH radiotherapy is also changing the research focus in this area. Accelerators that are used for VHEE can generally deliver radiation at ultra high dose rates, so that these accelerators are potentially very useful for FLASH dose delivery. This aspect is borne testimony to by the several recent studies on VHEE using UHDR [49], [50], [52]. It may well turn out that VHEE becomes a modality for UHDR delivery, rather than purely for its dose qualities.

The optimum energy of the electrons is currently still unclear. Several of the published studies have shown considerable benefit compared to photons at the energy of 100 to 200 MeV. Some researchers favour relatively low energies, since at these energies, there is a distinct peak in the depth dose curve at the depth of the PTV, but other researchers favour higher energies, due to the narrow penumbra and lack of scatter at these energies. There is a lack of studies in the UHEE energy range. Moreover, current knowledge may need to be revised in the light of further studies on focused beams.

One of the potential drawbacks of VHEE [56] is the possibility of increased neutron dose both in and around the patient, due to the bremsstrahlung photons undergoing photonuclear reactions, principally (γ,n), (γ,p), (γ,2n) and (γ,pn) reactions. The neutron dose was estimated by DesRosiers et al. [16] to be in the order of 0.2 % of the dose at the depth of maximum dose in the patient. With a quality factor of around 10, the neutrons were calculated to produce a biological effect in the order of 2 %, leading to a relative biological effectiveness of the VHEE beam of 1.02. However, more recently, Subiel et al. [57] used the FLUKA Monte Carlo code [24], [25] to calculate neutron flux in and around the electron beam and concluded that it is around 10^−5^ neutrons cm^−2^ per incident electron, which is negligible for practical purposes. This result was supported by a recent comprehensive study of Masilela et al. [2] using the TOPAS [22] Monte Carlo code. Their conclusion was that the protection required for this level of neutron radiation was no more significant than that required for proton treatment.

Choice of a broad beam or a pencil beam remains variable in the literature. A broad VHEE beam may be generated using the same technology as conventional low-energy electrons (5 – 20 MeV). However, this increases the photo-neutron production from the treatment machine compared to low-energy electrons. Using the spot-scanning technique, like contemporary proton treatment machines used for intensity-modulated proton therapy, allows the forming of a broad treatment field. Use of pencil beams requires practically that the beam is steered in a raster pattern over the target, possibly with simultaneously varying energy or energy layers. This provides a more exquisite dose distribution in general. There is also a possibility for arc treatment, rather like proton arc therapy or VMAT. However, application of VHEE to UHDR may require, at least initially, that broad beams are used, as they allow faster delivery of the treatment plan, so as to achieve a total delivery time for one fraction of less than 100 ms [49], [52].

The MM50 racetrack microtron offers some evidence for the applicability of the VHEE concept. Although the device did not succeed historically, it is interesting to conjecture what might be achieved in the modern radiotherapy environment. Equipped with a high-resolution multileaf collimator, a Monte Carlo treatment planning system and perhaps a higher energy of 100 MeV, could it have a role to play? Today’s radiotherapy community also has much more experience of advanced treatment planning, and proton therapy has provided experience of working with scanned pencil beams.

There are many types of technology available for the generation of VHEE beams [15], [58], [59], [60]. Among these, the PHASER device [54] appears to be promising for clinical application. The challenge of applying VHEE is to harness these technologies into producing stable and symmetrical beams with appropriate beam steering and accurate dosimetry and then to enclose the apparatus in an appropriate environment, so that clinical treatment can become a reality. Several companies are already in the process of developing viable clinical devices [61], [62], [63], [64]. However, treatment planning simulations are valuable for determining what is achievable from these technologies and how best to implement them.

## CRediT authorship contribution statement

**James L. Bedford:** Conceptualization, Writing – original draft. **Uwe Oelfke:** Supervision, Funding acquisition.

## Declaration of competing interest

The authors declare that they have no known competing financial interests or personal relationships that could have appeared to influence the work reported in this paper.
